# Author Correction: A fast approach for structural and evolutionary analysis based on energetic profile protein comparison

**DOI:** 10.1038/s41467-025-58565-0

**Published:** 2025-04-04

**Authors:** Peyman Choopanian, Jaan-Olle Andressoo, Mehdi Mirzaie

**Affiliations:** 1 Department of Pharmacology, Faculty of Medicine, University of Helsinki, Helsinki, Finland; 2 Division of Neurogeriatrics, Department of Neurobiology, Care Sciences and Society (NVS), Karolinska Institutet, Stockholm, Sweden

**Keywords:** Computational models, Protein analysis

Correction to: *Nature Communications* 10.1038/s41467-025-57374-9, published online 06 March 2025

In the version of the article initially published, Jaan-Olle Andressoo and Mehdi Mirzaie were not listed as having equally supervised the work and the sentence “Open access was supported by Helsinki University Library” was missing from the Acknowledgements section. These corrections have been made to the HTML and PDF versions of the article.

