# Supplementary material for: A fast approach for structural and evolutionary analysis based on energetic profile protein comparison
Source: Nat Commun. 2025 Mar 6;16:2231. doi: 10.1038/s41467-025-57374-9 (PMC11882786; doi:10.1038/s41467-025-57374-9)
Supplement: Supplementary file 2 — Reporting Summary [file 41467_2025_57374_MOESM2_ESM.pdf]

nature portfolio

Corresponding author(s): Mehdi Mirzaie
Jaan-Olle Andressoo

Last updated by author(s): 29.01.2025

# Reporting Summary

Nature Portfolio wishes to improve the reproducibility of the work that we publish. This form provides structure for consistency and transparency in reporting. For further information on Nature Portfolio policies, see our Editorial Policies and the Editorial Policy Checklist.

## Statistics

For all statistical analyses, confirm that the following items are present in the figure legend, table legend, main text, or Methods section.

| n/a | Confirmed | |
|---|---|---|
| [ ] | [x] | The exact sample size (*n*) for each experimental group/condition, given as a discrete number and unit of measurement |
| [x] | [ ] | A statement on whether measurements were taken from distinct samples or whether the same sample was measured repeatedly |
| [ ] | [x] | The statistical test(s) used AND whether they are one- or two-sided<br>*Only common tests should be described solely by name; describe more complex techniques in the Methods section.* |
| [x] | [ ] | A description of all covariates tested |
| [x] | [ ] | A description of any assumptions or corrections, such as tests of normality and adjustment for multiple comparisons |
| [ ] | [x] | A full description of the statistical parameters including central tendency (e.g. means) or other basic estimates (e.g. regression coefficient) AND variation (e.g. standard deviation) or associated estimates of uncertainty (e.g. confidence intervals) |
| [ ] | [x] | For null hypothesis testing, the test statistic (e.g. *F*, *t*, *r*) with confidence intervals, effect sizes, degrees of freedom and *P* value noted<br>*Give P values as exact values whenever suitable.* |
| [x] | [ ] | For Bayesian analysis, information on the choice of priors and Markov chain Monte Carlo settings |
| [x] | [ ] | For hierarchical and complex designs, identification of the appropriate level for tests and full reporting of outcomes |
| [x] | [ ] | Estimates of effect sizes (e.g. Cohen's *d*, Pearson's *r*), indicating how they were calculated |

*Our web collection on statistics for biologists contains articles on many of the points above.*

## Software and code

Policy information about availability of computer code

| | |
|---|---|
| Data collection | No software was used for data collection. |
| Data analysis | The following softwares were used for data analysis:<br>R version 4.4.2, and the packages including (Bio3D, readr, tidyverse, dplyr, tidyr, ggplot2, ggrepel, ggpubr, ggpointdensity, ggpubr, gridExtra, reshape2, umap, viridis, viridisLite, caret, randomForest, seqinr, geometry, ape, colorspace, dendextend, dynamicTreeCut, ggtree, phangorn, stringr, protr, proxy, class, readx, ggh4x, googledrive)<br>TM-Vec (Version v1), tmtools 0.1.1, MATLAB R2024a, SplitTree 4.19.2, Python 3.10.12, muscle3.8.1551<br><br>The custom codes and functions are available at<br>https://github.com/mirzaie-mehdi/ProteinEnergyProfileSimilarity |

For manuscripts utilizing custom algorithms or software that are central to the research but not yet described in published literature, software must be made available to editors and reviewers. We strongly encourage code deposition in a community repository (e.g. GitHub). See the Nature Portfolio guidelines for submitting code & software for further information.

# Data

Policy information about availability of data

All manuscripts must include a data availability statement. This statement should provide the following information, where applicable:

- Accession codes, unique identifiers, or web links for publicly available datasets
- A description of any restrictions on data availability
- For clinical datasets or third party data, please ensure that the statement adheres to our policy

1. The proteins used to train the knowledge-based potetnial function obtained from "Wang G, Dunbrack Jr RL. PISCES: a protein sequence culling server. Bioinformatics 19, 1589-1591 (2003)."

2. Astral 95/40 data downloaded form Scope 2.08 "https://scop.berkeley.edu/astral/"

3. The Bacteriocin proteins was obtained by requesting it from the authors of the paper 'Protein remote homology detection and structural alignment using deep learning'. This file contains results from AlphaFold2, OmegaFold, ESMFold, and TM-Vec software tools.

4. PDBIDs of Ferritin Superfamily (SCOP ID: a.25.1).

5. Two superfamilies protein domain IDs were downoladed form "https://www.cathdb.info/" (CATH Code: 1.10.8.10 and 3.10.28.10).

6. The list of protein domains of five superfamilies (Data/csv/fiveSF.csv) winged helix (SCOP ID: a.4.5), PH domain-like (SCOP ID: a.55.1), NTF-like (SCOP ID: d.17.4), Ubiquitin-like (SCOP ID: d.15.1), and Immunoglobulins (SCOP ID: b.1.1).

7. The Spike Glycoproteins in Covid dataset were downloaded from "https://cov3d.ibbr.umd.edu/"

8. The drug-target information downloaded from the supplementary information of the following paper "Cheng F, Kovács IA, Barabási A-L. Network-based prediction of drug combinations. Nature communications 10, 1197 (2019)".

9.The list of 21 mammalian hemoglobin's proteins in Globin family obtained from Supplementary Information of Freiberger MI, et al. Local energetic frustration conservation in protein families and superfamilies. Nature Communications 14, 8379 (2023).

10. Large-Scale SARS-CoV-2 Proteome Analysis across 28 families obtained from Supplementary Information of Freiberger MI, et al. Local energetic frustration conservation in protein families and superfamilies. Nature Communications 14, 8379 (2023).

All data used for our analysis is accessible at https://github.com/mirzaie-mehdi/ProteinEnergyProfileSimilarity/Data

# Research involving human participants, their data, or biological material

Policy information about studies with human participants or human data. See also policy information about sex, gender (identity/presentation), and sexual orientation and race, ethnicity and racism.

| | |
|---|---|
| Reporting on sex and gender | N/A |
| Reporting on race, ethnicity, or other socially relevant groupings | N/A |
| Population characteristics | N/A |
| Recruitment | N/A |
| Ethics oversight | N/A |

Note that full information on the approval of the study protocol must also be provided in the manuscript.

# Field-specific reporting

Please select the one below that is the best fit for your research. If you are not sure, read the appropriate sections before making your selection.

[x] Life sciences  [ ] Behavioural & social sciences  [ ] Ecological, evolutionary & environmental sciences

For a reference copy of the document with all sections, see nature.com/documents/nr-reporting-summary-flat.pdf

# Life sciences study design

All studies must disclose on these points even when the disclosure is negative.

Sample size

For statistical testing, the following samples sizes were used,
1. Comparison of CPE distances for all pairs of bacteriocins including, pairs at different classes (N=125869), pairs at the same class (N=111349), pairs from the same class from subclass1 (N=13431).
2. Within-group and between-group distance comparisons using CPE and SPE methods across different SCOP levels in the ASTRAL dataset.

ASTRAL40 Dataset:

• Within-group distances (Alpha class):
o n = 1000 piar domains from the Alpha class.
• Between-group distances (Alpha vs. other classes):
o n = 1000 Alpha-class pair domains compared with domains from other classes.
• Within-group distances (a.29 fold):
o n = 650 pair domains within the a.29 fold.
• Between-group distances (a.29 fold vs. other folds in all-alpha class):
o n = 3956 pair domains from other folds in the all-alpha class.
• Within-group distances (a.29.3 superfamily):
o n = 77 pair domains within the a.29.3 superfamily.
• Between-group distances (a.29.3 superfamily vs. other superfamilies in a.29 fold):
o n = 188 pair domains from other superfamilies within the a.29 fold.

ASTRAL95 Dataset:

• Within-group distances (Alpha class):
o n = 1000 pair domains from the Alpha class.
• Between-group distances (Alpha vs. other classes):
o n = 1000 Alpha-class pair domains compared with domains from other classes.
• Within-group distances (a.29 fold):
o n = 3872 pair domains within the a.29 fold.
• Between-group distances (a.29 fold vs. other folds in all-alpha class):
o n = 16,748 pair domains from other folds in the all-alpha class.
• Within-group distances (a.29.3 superfamily):
o n = 813 pair domains within the a.29.3 superfamily.
• Between-group distances (a.29.3 superfamily vs. other superfamilies in a.29 fold):
o n = 1344 pair domains from other superfamilies within the a.29 fold.
Generally, samples sizes are deemed sufficient for conducting systematic comparisons.

Data exclusions

A curated dataset of non-redundant protein chains was utilized using PISCES from the Protein Data Bank (PDB). The dataset was selected based on the following criteria:
• Pairwise sequence identity: Less than 50% to ensure non-redundancy.
• Resolution: Higher than 1.6 Å to guarantee structural accuracy.
• R-factor: Below 0.25 to ensure reliable crystallographic data.
• Protein length: Between 40 and 1,000 residues to include proteins of varying sizes while excluding excessively short or long chains.
• Overlap: Proteins overlapping with the test sets from this manuscript were removed from the training set.

Replication

This study outlines several measures to verify the reproducibility of the experimental findings, including:
1. Use of Benchmark Datasets: The study employed well-established datasets like ASTRAL40 and ASTRAL95 from SCOPe, ensuring that the findings were tested against widely recognized and reliable data sources. These datasets are filtered for specific sequence identity thresholds and structural similarity scores, enhancing reproducibility.
2. Correlation Analysis: High correlation coefficients between energy profiles derived from protein sequences and structures were reported. This suggests consistency in results across different levels of analysis, supporting reproducibility.
3. Cross-Validation: For classification tasks, the study utilized Leave-One-Out Cross-Validation (LOOCV) approach with 1-NN classifiers. This method enhances the reliability of the results by ensuring the model performs consistently across different subsets of data.
4. Comparison with Other Methods: The performance of the proposed methods (CPE and SPE) was compared with existing tools such as TM-Vec, RMSD, and TM-Score. Superior accuracy and computational efficiency were demonstrated, validating the method against established benchmarks.
5. Phylogenetic Reconstruction: The study successfully reconstructed phylogenetic trees using energy profiles, with results aligning with prior investigations. This consistency supports the robustness of the approach.
6. Clustering Analysis: Clustering of spike glycoproteins and bacteriocins based on energy profiles provided clear and reproducible groupings, corroborated by Adjusted Rand Index (ARI) metrics, which confirmed the clustering accuracy.
Replication Success: The article does not explicitly mention unsuccessful replication attempts or findings that cannot be reproduced. All methods and results appear to have been reproducible within the study, as demonstrated by consistent results across datasets and alignment with previously reported findings.

Randomization

The grouping and analysis methods used in the study were determined by the inherent structure of the datasets and the objectives of the

computational approaches. Here is how the samples were handled:
1. Pre-Labeled Datasets:
o The datasets used in the study (e.g., ASTRAL40, ASTRAL95, BAGEL) are curated resources with established classifications of protein domains based on structural and sequence-based criteria. These datasets inherently include groups based on classes, folds, superfamilies, or families, which served as the experimental groups for the study.
2. Classification Tasks:
o For classification experiments, protein domains were grouped into their respective hierarchical levels (e.g., folds, superfamilies, and families) as defined by SCOP or BAGEL annotations. These pre-existing classifications served as the experimental groups.
3. Clustering Analysis:
o For clustering tasks, samples (e.g., SARS-CoV, SARS-CoV-2, and MERS-CoV spike glycoproteins) were allocated into groups based on the calculated distances between energy profiles. The method was unsupervised, meaning the allocation into clusters was a result of the computational analysis rather than predetermined grouping.
Relevance of Non-Random Allocation:
Random allocation was not relevant to this study because:
• The groups (e.g., protein classes, superfamilies, or families) were determined by the inherent structure of the datasets.
• The study's focus was on computational methods for analyzing energy profiles, which rely on the characteristics of the data rather than experimental manipulation.

Blinding

Blinding was not relevant for this study due to its reliance on computational techniques and publicly available datasets which consist of pre-labeled and well-documented protein classifications. These datasets do not involve subjective evaluations that could be influenced by investigator bias. On the other hand, this study employs automated methods, such as energy profile calculation, machine learning classifiers (e.g. 1-NN), and clustering techniques. These computational approaches do not depend on manual categorization or subjective input.

# Reporting for specific materials, systems and methods

We require information from authors about some types of materials, experimental systems and methods used in many studies. Here, indicate whether each material, system or method listed is relevant to your study. If you are not sure if a list item applies to your research, read the appropriate section before selecting a response.

## Materials & experimental systems

| n/a | Involved in the study |
|---|---|
| ☒ | ☐ Antibodies |
| ☒ | ☐ Eukaryotic cell lines |
| ☒ | ☐ Palaeontology and archaeology |
| ☒ | ☐ Animals and other organisms |
| ☒ | ☐ Clinical data |
| ☒ | ☐ Dual use research of concern |
| ☒ | ☐ Plants |

## Methods

| n/a | Involved in the study |
|---|---|
| ☒ | ☐ ChIP-seq |
| ☒ | ☐ Flow cytometry |
| ☒ | ☐ MRI-based neuroimaging |

## Plants

Seed stocks: N/A

Novel plant genotypes: N/A

Authentication: N/A
